# Retention of a Foreign Body in the Vagina of an Adult for 13 Years: A Case Report

**DOI:** 10.7759/cureus.34776

**Published:** 2023-02-08

**Authors:** Omar M Sharaf, Elizabeth A Wilkinson, Elizabeth Elbadri, Emily E Weber LeBrun

**Affiliations:** 1 College of Medicine, University of Florida, Gainesville, USA; 2 Division of Female Pelvic Medicine and Reconstructive Surgery, Department of Obstetrics and Gynecology, University of Florida Health, Gainesville, USA

**Keywords:** duplicated ureter, vaginal foreign body, mullerian anomaly, transverse vaginal septum, chronic pelvic pain

## Abstract

Cases in which foreign bodies have been inserted into the vagina predominately occur in the pediatric population. This report presents the case of an adult woman with a retained foreign body for 13 years. A duplicated ureter suggestive of a Mullerian anomaly was incidentally identified on intraoperative cystoscopy. Mullerian anomalies may be associated with complex patient presentations and are associated with reproductive implications that should be discussed based on patient-specific characteristics.

## Introduction

Vaginal foreign bodies include a wide variety of objects depending on age group and may lead to complications such as pelvic inflammation, necrosis with fistula formation, vaginal stenosis, or toxic shock syndrome [1]. Most cases of vaginal foreign bodies are reported in pediatrics although some cases have been reported in adults. Symptoms primarily include malodorous, purulent blood-stained vaginal discharge, and lower abdominal pain with menses [2]. Patients may not remember foreign body insertion [3]; thus, vaginal foreign bodies often go undetected for long periods of time, up to 20 years in one case [4]. In cases where routine pelvic examination does not identify a vaginal foreign body, imaging with X-ray and/or pelvic ultrasound may be performed, but these objects are best detected with magnetic resonance imaging (MRI) [5]. Surgical removal is typically successful with fibrosis and vaginal stenosis commonly present at the time of diagnosis [1]. We present a case of an adult female with chronic pelvic pain who had a vaginal foreign body undetected for 13 years. This paper was previously presented at the American Urogynecologic Society 41st Annual Meeting in Vancouver, Canada, in 2020.

## Case presentation

The University of Florida does not require Institutional Review Board approval for case reports. Patient informed consent was obtained. A 31-year-old, gravida zero female presented to the clinic with chronic pelvic pain for several years. She had a menstrual history of regular cycles every month, and she had never been sexually active. The patient reported a history of using tampons while she was in high school for menstrual control; however, she had used pads ever since due to malodorous discharge of unknown etiology. She reported that she had been getting yearly gynecologic exams and had reportedly normal pap smears at another institution although there was no record of this. The patient’s pelvic pain was thought to be related to endometriosis, and she had previously been started on oral contraceptive medication.

**Figure 1:**
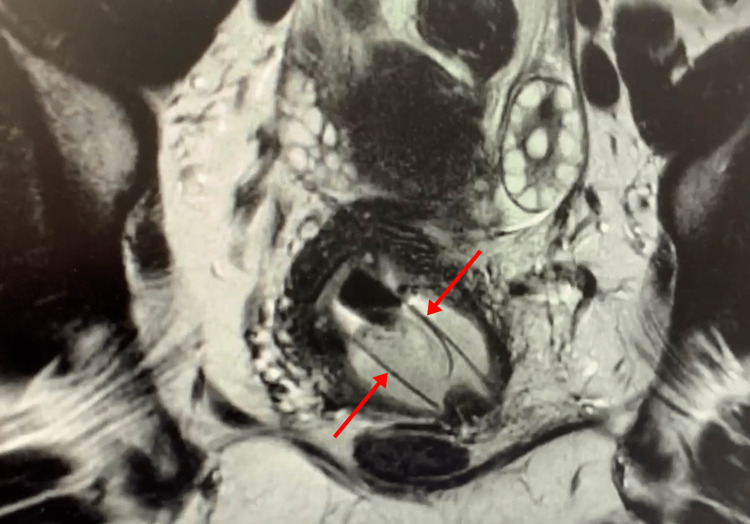
MRI displaying the anatomy of the cervix and upper vagina: a tubular foreign body is visualized within the endocervical canal and upper vagina. MRI, magnetic resonance imaging

On speculum examination at our institution, an obstruction was noted in the upper one-third of the vagina, obscuring the cervix. A bimanual examination revealed anterior/posterior fibrotic-like adhesions. Transvaginal ultrasound was ordered for imaging and showed distorted anatomy with no identifiable cervix. An MRI was obtained to further define the anatomy and demonstrated a 5 cm × 2 cm tubular foreign body positioned obliquely within the endocervical canal and upper vagina with surrounding fluid (Figure 1). Based on the size and shape of the foreign object, it was suspected to be a plastic tampon applicator that had been in place for 13 years as the patient had not used tampons since high school.

**Figure 2:**
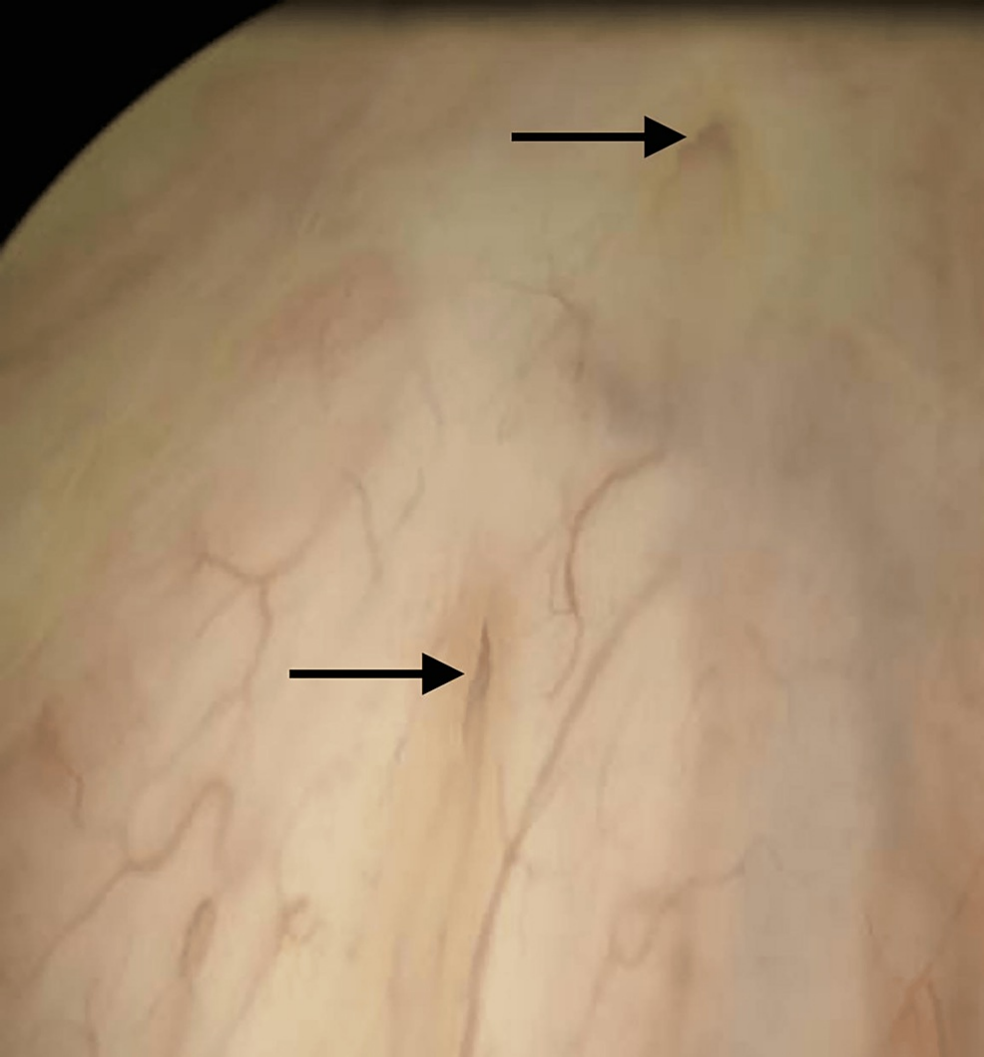
Intraoperative cystoscopy; a duplicated left ureter identified intraoperatively.

**Figure 3:**
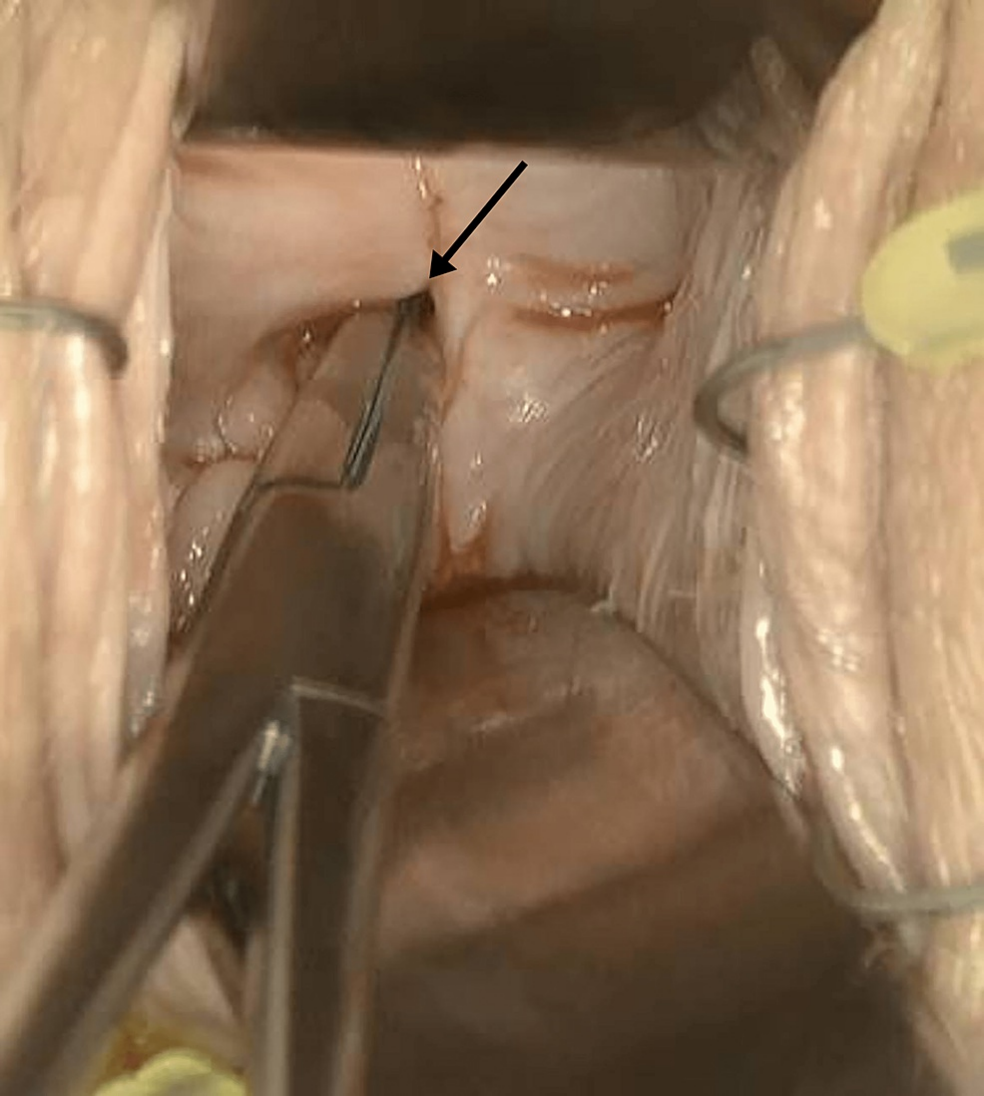
Circumferentially stenosed vaginal canal: a small dimple could be probed with a right-angle clamp.

**Figure 4:**
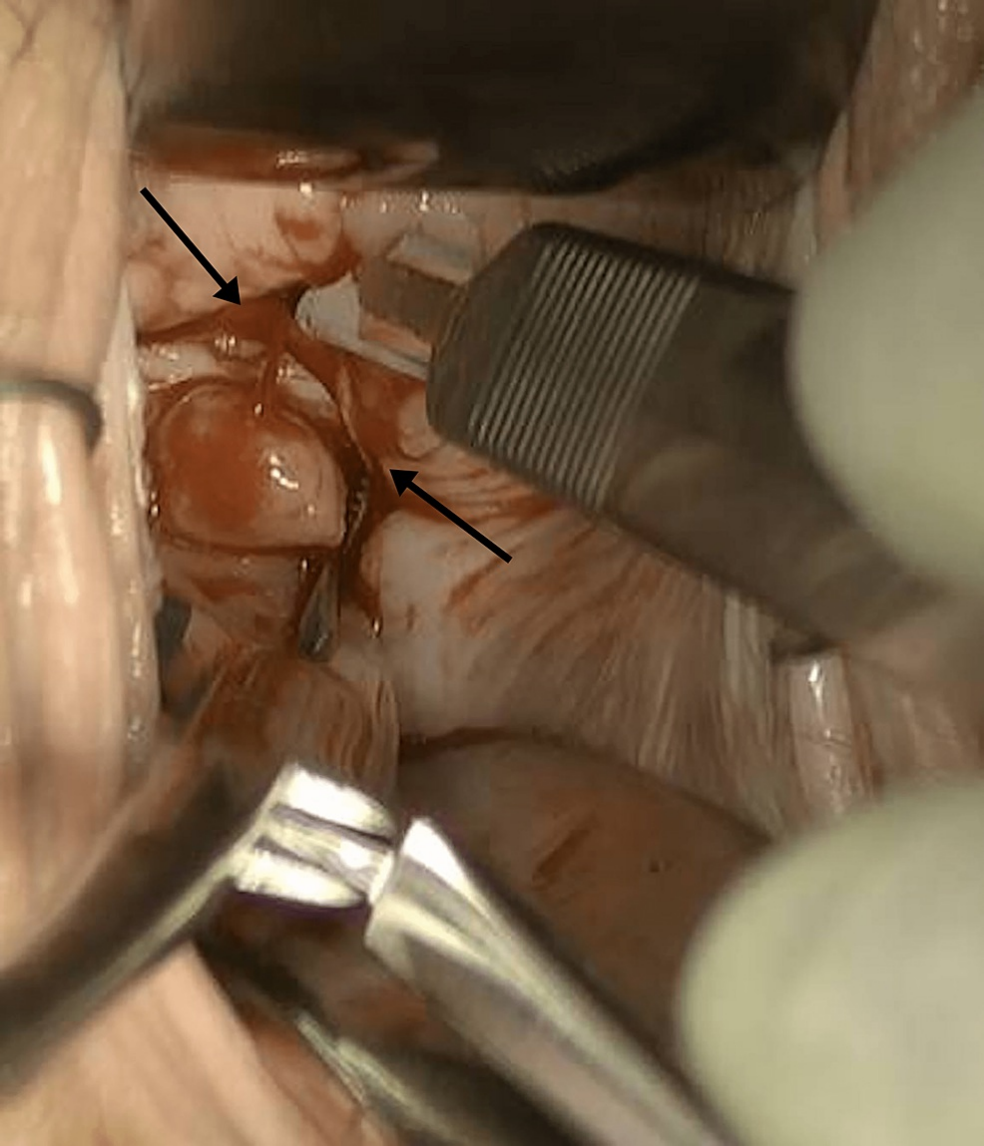
Incising the stenosed vaginal canal: a scar was incised with a 15-blade scalpel, while spreading tissue with a right-angle clamp; expression of purulent fluid.

**Figure 5:**
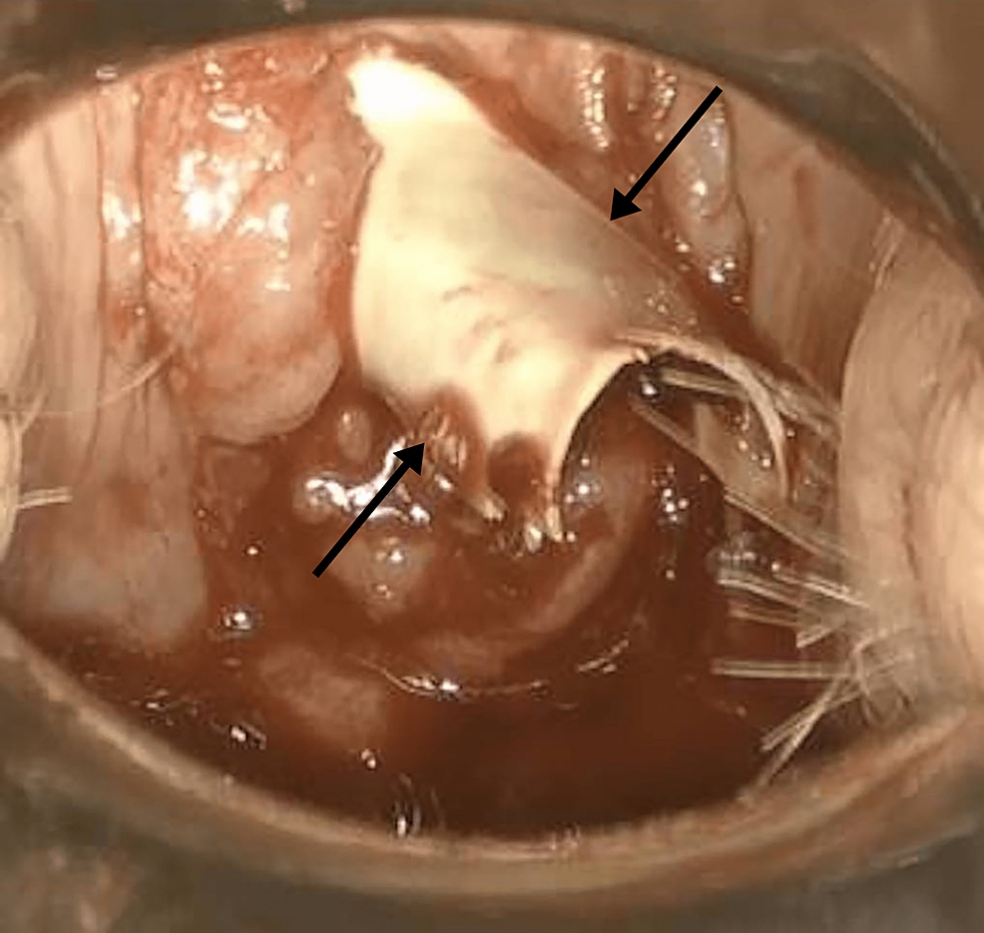
Tampon applicator removal: the opening was further stretched and incised, eventually allowing the removal of the plastic tampon applicator.

**Figure 6:**
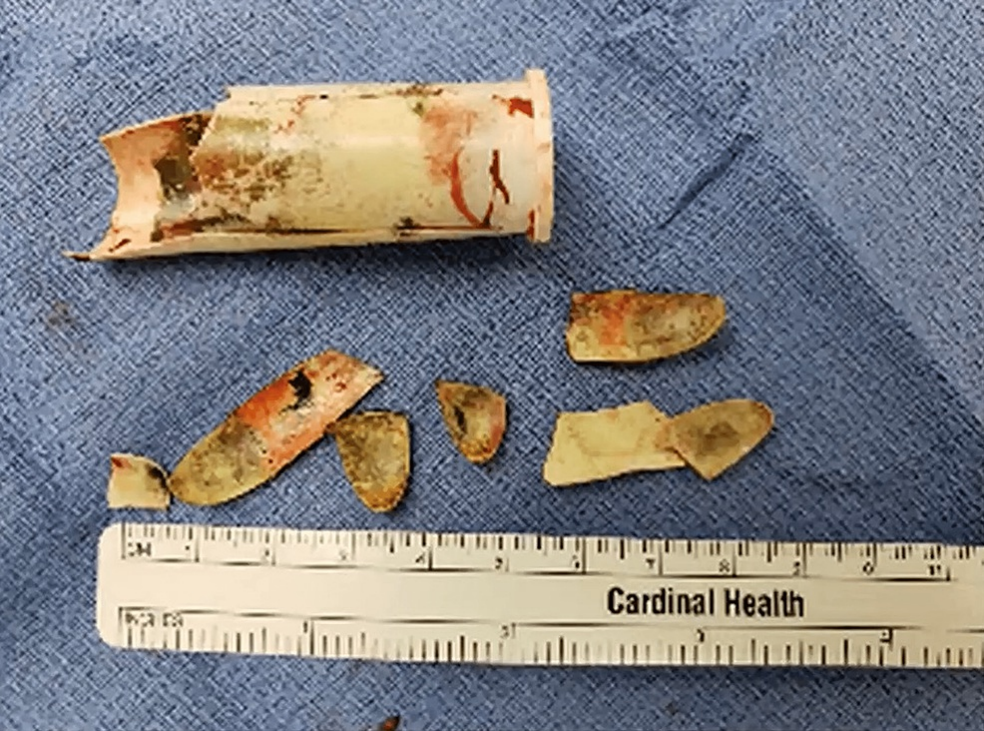
A plastic tampon applicator broken into eight segments.

The patient went to the operating room for removal. Cystoscopy was performed first and was significant for an incidental finding of a duplicated ureter on the left side (Figure 2). On examination under anesthesia, the patient had near-complete obliteration of the vaginal canal with circumferential vaginal stenosis with a vertical midline scar at the apex. There was a small dimple at the base of the scar that was probed cephalad demonstrating a patent canal (Figure 3). A vertical incision was made, and further probing expressed purulent fluid (Figure 4). A 30 cc Foley was placed through the narrowed opening and into the cavity. The Foley assisted with the direction of the additional dissection. The foreign body was visualized and grasped with a Babcock clamp and gently removed (Figure 5). Care was taken to prevent fragmentation of the specimen. A repeat cystoscopy was performed and noted no injury to the bladder. Vaginoscopy was performed, and additional pieces of foreign material were removed with cystoscopic graspers. The cervix itself could not be discerned from the surrounding inflamed and excoriated tissue. No fistula tract was noted. Once open, the vaginal canal measured 15 cm from the hymen to the cervix. The final specimen was a cylindrical plastic tube measuring 1 cm × 5 cm and broken into eight pieces, consistent with a plastic tampon applicator (Figure 6).

## Discussion

Most cases of vaginal foreign bodies are reported in the pediatric population. In a retrospective review of 35 girls aged 2.6 to 9.2 years with vaginal foreign bodies, predominant symptoms included vaginal bleeding and blood-stained, foul-smelling vaginal discharge [2]. Additionally, 46% of the patients did not recall the insertion of the foreign object [2]. The duration between the onset of symptoms to diagnosis varied from one day to two years in this pediatric population [2]. In adult women, there are a few case reports of vaginal foreign bodies. In a case series, two adult patients aged 34 and 44 years with a known history of vaginal foreign body insertion developed symptoms of vaginal discharge and lower abdominal discomfort with identification of the item seven days and two weeks after entry, respectively, via pelvic examination, X-ray, and abdominal ultrasound [6]. Chopra et al. reported on another case of a vaginal foreign body in a 50-year-old postmenopausal woman, with no recollection of foreign body insertion. She presented with foul-smelling, blood-stained vaginal discharge for three months [7]. The pelvic examination, X-ray, and pelvic ultrasound identified the foreign body, and surgical removal was successful without complications [7]. These reports suggest that recollection of vaginal foreign body insertion may be associated with more prompt identification and removal, as would be expected. However, given the frequency of cases in which a patient does not recollect foreign body insertion, it is necessary to consider the vaginal foreign body in patients with vaginal symptoms or abdominal discomfort with no known history of foreign body insertion. Timely recognition and removal of vaginal foreign bodies are necessary because undetected vaginal foreign bodies are associated with morbidity, which may include infertility, toxic shock syndrome, or fistula formation [1].

In the present case, a 31-year-old female presented with chronic pelvic pain and normal menstrual cycles. She was misdiagnosed with endometriosis based on clinical symptoms while a thorough cervical/pelvic evaluation was incomplete. After unsuccessful treatment with oral contraceptives, the pelvic examination and transvaginal ultrasound revealed distorted pelvic anatomy. MRI revealed a vaginal foreign body, although the patient had no recollection of foreign body insertion. The foreign body was surgically removed, during which intraoperative cystoscopy revealed a duplicated ureter.

There is a well-recognized association between renal malformations and Mullerian anomalies [8]. The duplicated ureter suggested a potentially coexistent Mullerian anomaly. Given the patient’s pelvic exam and intraoperative findings, this patient likely has a Mullerian anomaly consistent with an incomplete transverse vaginal septum. An undiagnosed Mullerian anomaly may have predisposed this patient to long-term foreign body retention and complex surgical removal. To our knowledge, this is the first reported case in the literature of a patient with a transverse vaginal septum and retained vaginal foreign body. The finding of a duplicated ureter suggesting a coexistent Mullerian anomaly additionally makes this case unique.

This case highlights the need to evaluate for vaginal foreign bodies in adult women who present with malodorous vaginal discharge or chronic pelvic pain, even when they have no recollection of foreign body insertion. Although X-ray and pelvic ultrasound may detect vaginal foreign bodies, MRI provides the best visualization and may facilitate complete removal. A vaginal surgical approach was appropriate for this patient to access and remove the retained foreign body. In cases where the transverse septum is too thick to dilate, a laparoscopic approach with posterior colpotomy and removal of the foreign body using an Endo-Catch bag may be a reasonable approach. When present concomitantly with a vaginal foreign body, the transverse vaginal septum should be addressed during foreign body removal as vaginal foreign bodies are associated with significant morbidity [1]. However, when present in isolation, there are important reproductive implications to consider when deciding whether to resect a transverse vaginal septum.

In a reproductive-aged individual in whom a Mullerian anomaly is suspected, it is important to counsel the patient regarding the impact of this diagnosis on reproduction. A transverse vaginal septum is associated with infertility and the arrest of labor [9]. Although surgical management has improved with modern techniques, resection of the transverse vaginal septum is still associated with restenosis and hematocolpos [10]. Thus, surgical resection is best performed in patients who are committed to postoperative therapies such as vaginal dilation. Patients' reproductive plans should be discussed to guide this decision. If a patient desires future childbearing, it would be important to determine her ideal family number. If she desires a small family, she may benefit from primary cesarean section rather than surgical septum resection and subsequent dilation therapy. However, in a patient who has a desire for a large family, there could be significant benefit from surgical septum resection compared to repeat cesarean section, given the subsequent risk of invasive placenta with each cesarean section. Although the presence of a vaginal foreign body in the current patient was an indication for operative removal with concomitant transverse vaginal septum resection, this highlights an additional consideration in patients with isolated Mullerian anomalies, and management recommendations may differ based on individual patient circumstances.

## Conclusions

Retained vaginal foreign bodies in adult patients are rare and often overlooked. Patients frequently do not recall foreign body insertion but may present with malodorous vaginal discharge and/or abdominal pain. Vaginal foreign bodies are best visualized with MRI and should be removed promptly to avoid morbidity. Vaginal foreign body removal may be achieved with vaginal or laparoscopic surgical approaches depending on intraoperative findings. Mullerian anomalies may be associated with complex patient presentations and are associated with reproductive implications that should be discussed based on patient-specific characteristics.
